# Methanol Oxidation at Platinum Coated Black Titania Nanotubes and Titanium Felt Electrodes

**DOI:** 10.3390/molecules27196382

**Published:** 2022-09-27

**Authors:** Aikaterini Touni, Xin Liu, Xiaolan Kang, Chrysanthi Papoulia, Eleni Pavlidou, Dimitra Lambropoulou, Mihalis N. Tsampas, Athanasios Chatzitakis, Sotiris Sotiropoulos

**Affiliations:** 1 Department of Chemistry, Aristotle University of Thessaloniki, 54124 Thessaloniki, Greece; 2 Centre for Materials Science and Nanotechnology, Department of Chemistry, University of Oslo, Gaustadalléen 21, 0349 Oslo, Norway; 3 Department of Physics, Aristotle University of Thessaloniki, 54124 Thessaloniki, Greece; 4 Dutch Institute for Fundamental Energy Research (DIFFER), 5612 AJ Eindhoven, The Netherlands

**Keywords:** methanol oxidation, galvanic deposition, titanium, titania nanotubes, black titania

## Abstract

Optimized Pt-based methanol oxidation reaction (MOR) anodes are essential for commercial direct methanol fuel cells (DMFCs) and methanol electrolyzers for hydrogen production. High surface area Ti supports are known to increase Pt catalytic activity and utilization. Pt has been deposited on black titania nanotubes (bTNTs), Ti felts and, for comparison, Ti foils by a galvanic deposition process, whereby Pt(IV) from a chloroplatinate solution is spontaneously reduced to metallic Pt (at 65 °C) onto chemically reduced (by CaH_2_) TNTs (resulting in bTNTs), chemically etched (HCl + NaF) Ti felts and grinded Ti foils. All Pt/Ti-based electrodes prepared by this method showed enhanced intrinsic catalytic activity towards MOR when compared to Pt and other Pt/Ti-based catalysts. The very high/high mass specific activity of Pt/bTNTs (ca 700 mA mg_Pt_^−1^ at the voltammetric peak of 5 mV s^−1^ in 0.5 M MeOH) and of Pt/Ti-felt (ca 60 mA mg_Pt_^−1^, accordingly) make these electrodes good candidates for MOR anodes and/or reactive Gas Diffusion Layer Electrodes (GDLEs) in DMFCs and/or methanol electrolysis cells.

## 1. Introduction

Electrochemistry is expected to play a key role in the carbon-neutral transition, since it can offer several pathways to produce and store energy and thus reduce the carbon footprint of the mobility, domestic and industrial sectors [1]. Among the various electrocatalytic systems, methanol oxidation reaction (MOR) has received much attention due to its multifaceted scientific and technological importance [2,3].

First, methanol is considered as a promising alternative fuel because it offers high energy density, low production cost and, unlike hydrogen, it can be easily handled, transported and stored due to its liquid form [4,5,6,7,8,9,10,11,12]. The chemical energy of methanol can be converted to electrical energy within a direct methanol fuel cell (DMFC), where MOR takes place at the anode. DMFCs have attracted interest mainly for their applications to small portable devices. Second, it is well established that alcohols can significantly reduce the energy demands of water electrolysis via a process known as alcohol electrolysis, or alcohol electro-reforming or chemical-assisted hydrogen evolution [13,14,15,16,17,18,19,20]. In this context, methanol can potentially play a role in the efficient storage of renewable electricity into hydrogen. In essence, the concept lies in replacing the thermodynamically demanding (1.23 V onset potential) oxygen evolution reaction by a reaction with lower thermodynamic demands, such as MOR (0.016 V onset potential). Finally, MOR is a reaction of high scientific importance. Due to its simplicity, since it does not involve the breaking of C-C bonds, MOR serves as a model system to provide fundamental understanding of the interactions between electrocatalysts and longer chain alcohols [21,22].

Investigations on various MOR catalysts have shown that excellent catalytic activity can only be obtained with Pt-based materials [2,11]. The main associated challenges with these catalysts are the high cost of Pt and the poor durability due to poisoning species by surface-bonded carbon monoxide [23,24]. To address these challenges, efforts are mainly directed either towards minimizing Pt loadings via the use of binary or ternary catalysts [25,26,27,28,29] or towards increasing Pt utilization by using advanced nanostructures and by maximizing dispersion via the use of highly porous supports [3]. Bimetallic PtRu is considered as the most active catalyst due to its bifunctional mechanism and the ligand effect. Hence, PtRu has become an interesting catalyst alloy and has been used until today with many carbon supports. However, the toxicological effect of the addition of ruthenium (Ru) metal remains uncertain [29].

In fuel cells or electrolyzers, the electrocatalysts (either supported or unsupported) should be used in connection with porous substrates that form gas diffusion layer electrodes (GDLEs) [30]. Carbon-based supports and substrates which are typically used in hydrogen fuel cells are prone to corrosion at the MOR operating potentials [24,29]. Thus, Ti-based supports and substrates are more appropriate for MOR electrodes.

Ti or TiO_2_ supports can address both of the above challenges since they can be used in high surface area forms (Ti meshes [31,32], TiO_2_ porous layers [33], nanoparticles [34,35,36] or nanotubes (TNTs) [37,38,39,40,41,42,43]); both Ti and TiO_2_ are known at the same time to promote MOR at Pt [31,32,33,34,35,36,37,38,39,40,41,42,43,44,45,46,47,48]. This is attributed to either altering the electron density on Pt and affecting the Pt-CO bonding or, primarily, to Ti or reduced/defective TiO_2-x_ sites exhibiting an oxophilic character that promotes the activation of water to OH_ads_ species which in turn react with/regenerate Pt-CO species/sites (bifunctional mechanism [27,45,49]). Electron conductivity is also an important functionality for the support and substrate materials. To deal with the low conductivity of TiO_2_ supports and thus improve electron transport through the electrode, several strategies can be followed such as increasing the Pt content, doping/combining with C [37,40,43], increasing the number of O vacancies and Ti^3+^ defects by reductive treatment, either chemical (often leading to titania black-type of materials [50]) or electrochemical [41].

There are various methods for Pt deposition on titanium/titania substrates and these include chemical/hydrothermal/photochemical methods [33,34,35,36] (usually employed in the case of nanoparticle supports) and atomic layer deposition, ALD [42,47,51] as well as electrodeposition methods [37,38,39,40,41,43,44,45,46,48] (preferred for Ti and TNT substrates). An alternative method for deposition on Ti and TNT supports is that of spontaneous galvanic deposition of Pt from Pt(IV) complex solutions onto freshly etched/reduced Ti and TNTs. The method is a simple two-step process (etch/reduce and deposit) that has already been used successfully for the fabrication of Pt/Ti [52,53] and Ir/Ti [54] as well as Pt/black TNTs (bTNTs) [50]. The driving force of the overall reaction is the difference in thermodynamic redox potentials of two-half cell reactions: Pt(IV) is reduced to Pt(0) on the substrate surface taking up electrons from reductive surface species (Ti(0) [52,53], Ti(III) [54] or trapped e^−^ in the presence of oxygen vacancies [50]), which in turn get oxidized/are annihilated. To the best of our knowledge, MOR has not been studied on Pt-coated Ti felts or bTNT electrodes prepared by galvanic deposition.

The aim of this work has been the development of a novel type of Pt electrocatalysts supported on high surface area titanium/titania supports that may find applications as efficient MOR anodes and modified GDLEs in direct methanol fuel cells and methanol electrolysis for hydrogen production. Its objectives have been: (a) the preparation by means of galvanic deposition o Pt deposits onto/into titania black nanotubes (Pt/bTNT) and their microscopic characterization, (b) the preparation and characterization in a similar manner of Pt coatings on Ti felt supports (Pt/Ti-f) and (c) testing both electrode systems as anodes for MOR by means of voltammetry.

## 2. Results

### 2.1. Microscopic (SEM) and Spectroscopic (EDS, ICP-MS) Characterization

**Figure 1:**
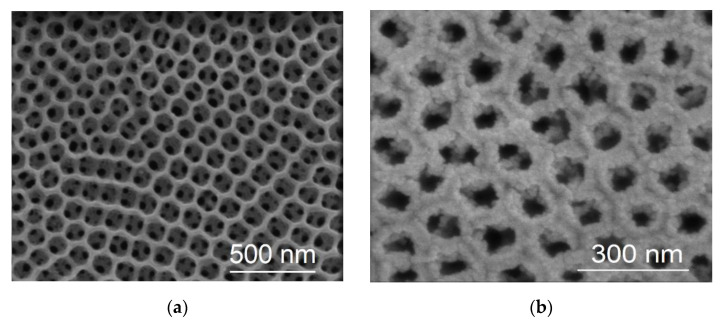
Top-view SEM micrographs of (**a**) bTNTs and (**b**) Pt/bTNTs. (Figure S1: X-ray diffractogram of Pt/bTNT that includes peaks from TiO2 in anatase structure, hexagonal Ti and cubic Pt according to the corresonding ICDD-JCPD files. As it can be observed, the Ti and Pt characteristic peaks are present in the spectrum of Pt/bTNT).

Figure 1a shows a top SEM view of a bTNT substrate prepared by anodization for 5 min (bTNT5), followed by high temperature reduction by CaH_2_ and annealing (for exact procedure see Section 4.1 in Materials and Methods as well as Ref. [33]). Nanotubes of 2.03 μm length and a ca 200 nm diameter, with a long-range open structure have been obtained; the latter is important both for platinization of the interior of the nanotubes (via unrestricted access of the chloroplatinate reactant to the interior of the structure) as well as limited CO_2_ clogging during their use as MOR anodes. Indeed, close inspection of Figure 1b that depicts the SEM micrograph of the platinized Pt/bTNT electrode, reveals that, apart from complete coverage of the rims of the outer surface of the nanotubes by a nodular Pt deposit, Pt is also deposited inside the pores. (Similarly prepared systems, reported in our previous work [50], consist of Pt nanoparticles with an average size of 2.5 nm that tend to form aggregates with a 10–30 nm diameter). Analysis of the acid (aqua regia)-etched deposits by ICP-MS, resulted in the determination of a 19 μg_Pt_ cm_geom_^−2^ Pt loading.

**Figure 2:**
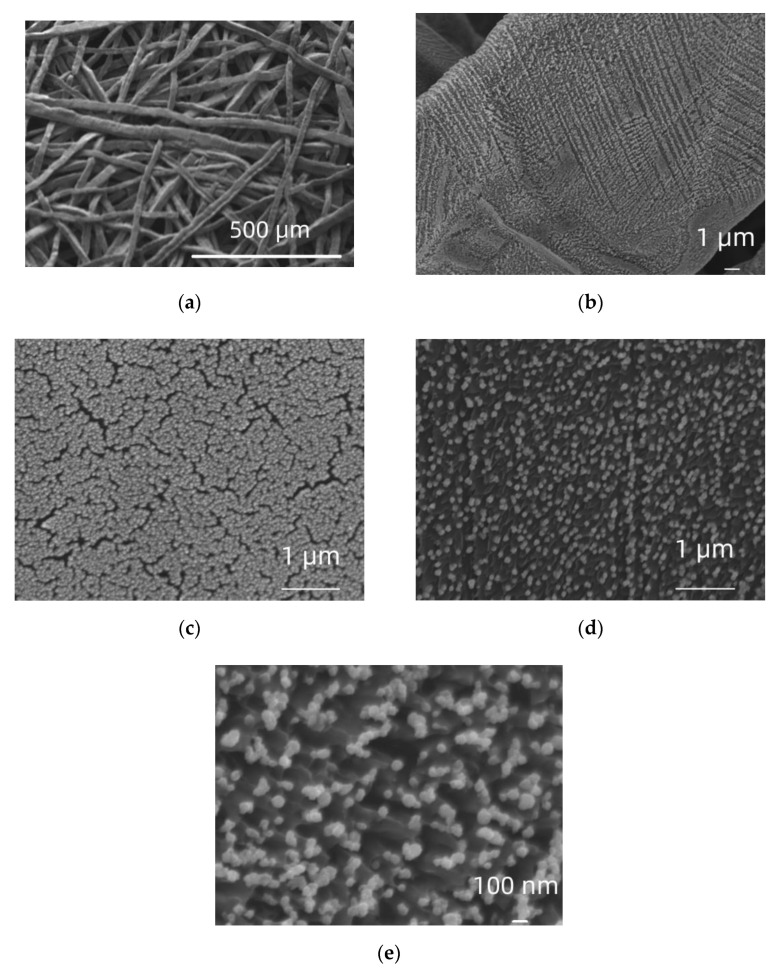
SEM micrographs of a Pt/Ti felt at a magnification of ×100 (**a**), ×4.5 k (**b**), ×20 k (**c**) + (**d**) and ×50 k (**e**).

Figure 2a–e depict SEM micrographs of a Ti felt after platinization by galvanic deposition (denoted as Pt/Ti. It can be seen that, although Pt was deposited throughout the Ti network (as confirmed by micrographs at various locations, the density of deposited metal varies (see difference between locations shown in (b), (c) and (d)) and so does its morphology, ranging from closely packed stripes and patches to nanoparticles. At higher resolution aggregated particles with diameter of approximately 60–100 nm are observed (Figure 2e at magnification of 50 k). EDS analysis of areas of (b) give approximately a 3.7% Pt atomic percentage. The uneven deposition at microscopic level results in a moderate (for a high surface area support) Pt loading of 65 μg_Pt_ cm_geom_^−2^ (per nominal, projected mesh area).

**Figure 3:**
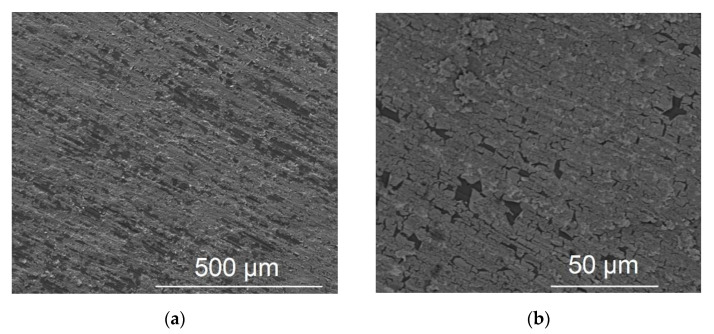
SEM micrographs of Pt/Ti foil: (**a**) SEI at a magnification of ×150 and (**b**) BEI at a magnification of ×1 k.

Figure 3a,b depict the SEM micrographs of Pt galvanic deposits on a freshly grinded Ti foil. One can see that almost complete surface coverage is achieved with only a few uncovered spots present; this is clearly shown in Figure 3b where the backscattered electron image reveals a white overlayer corresponding to the Pt film, disrupted by some black spots corresponding to uncovered Ti. EDS measurements give an indicative 9.7% Pt–90.3% Ti atomic composition. An accurate estimate of the Pt loading, by means of ICP-MS analysis of the etched deposit, gives a value of 214 μg_Pt_ cm_geom_^−2^.

### 2.2. Surface Electrochemistry in Acid

**Figure 4:**
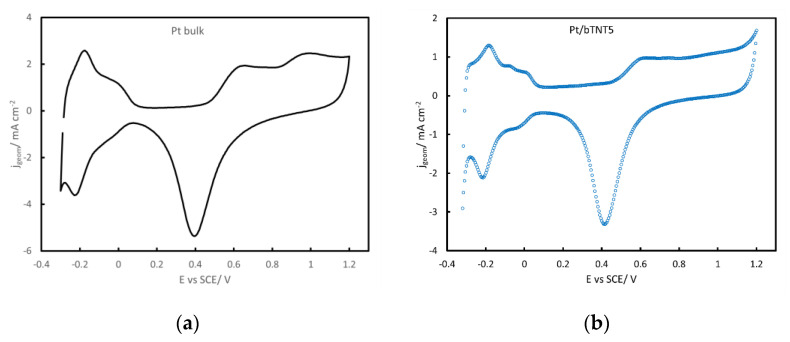

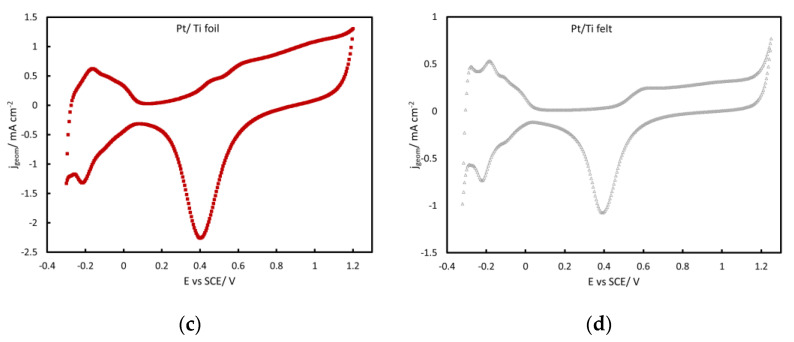
Cyclic voltammograms of (**a**) polycrystalline bulk Pt at a scan rate of 500 mV s^−1^, (**b**) Pt/bTNT5 at 50 mV s^−1^, (**c**) Pt/Ti foil at 50 mV s^−1^ and (**d**) Pt/Ti felt at 50 mV s^−1^, in a N_2_-deaerated 0.1 M HClO_4_ solution.

As can be seen from the cyclic voltammograms of Figure 4 below, all platinized Ti electrodes reveal features characteristic of Pt surface electrochemistry: reversible UPD-H (underpotentially deposited hydrogen) peaks in the −0.3–+ 0.1 V_SCE_ potential range, a double layer (“flat current”) region at potentials more positive than +0.1 V_SCE_ and the Pt oxide formation/reduction regions (as a current wave starting at ca. +0.5 V_SCE_ and reaching a plateau during the anodic scan and a peak at ca. +0.4 V_SCE_ during the cathodic scan respectively). Pt electroactive areas, estimated from the charge calculated by integrating the anodic peaks of UPD- desorption in the −0.3–+0.1 V_SCE_ range (assuming 210 μC cm^−2^ [55]), were 30.51 cm_Pt_^2^ cm_geom_^−2^ for Pt/bTNT5, 14.88 cm_Pt_^2^ cm_geom_^−2^ for Pt/Ti foil, 8.95 cm_Pt_^2^ cm_geom_^−2^ for Pt/Ti felt and 5.16 cm_Pt_^2^ cm_geom_^−2^ for abraded polycrystalline bulk Pt.

### 2.3. MOR in Acid

**Figure 5:**
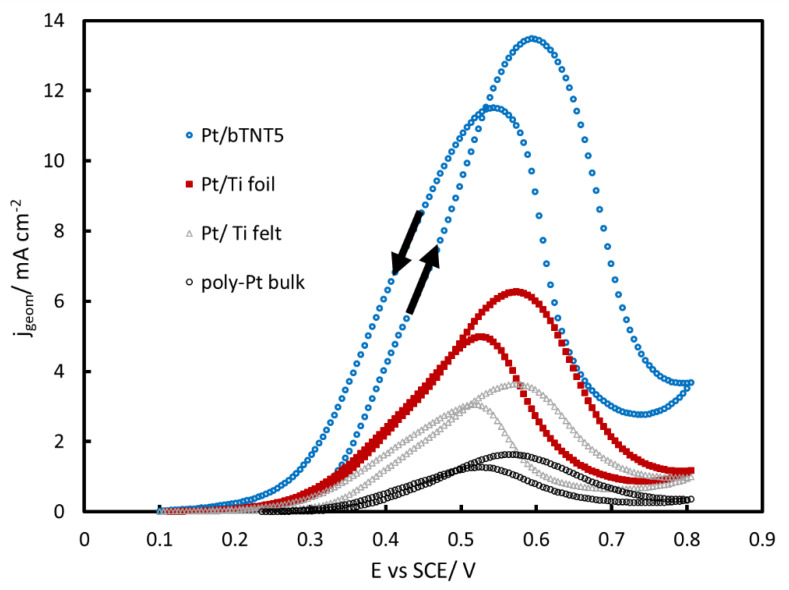
Voltammograms of Pt/bTNT5, Pt/Ti foil, Pt/Ti felt and polycrystalline bulk Pt in a N_2_-deaerated solution of 0.1 M HclO_4_ + 0.5 M MeOH, at a low scan rate of 5 mV s^−1^, scanned between +0.1–+0.8 V_SCE_ (forward and backward scans). The current is normalized per substrate geometric area in cm^2^.

**Figure 6:**
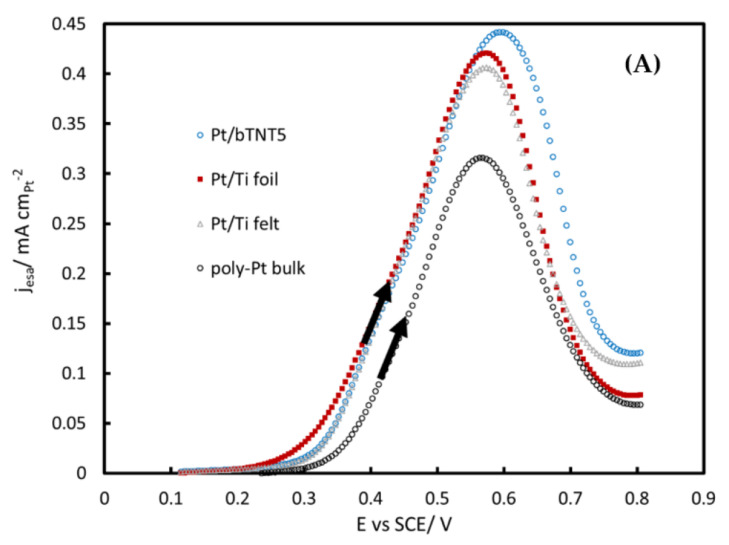

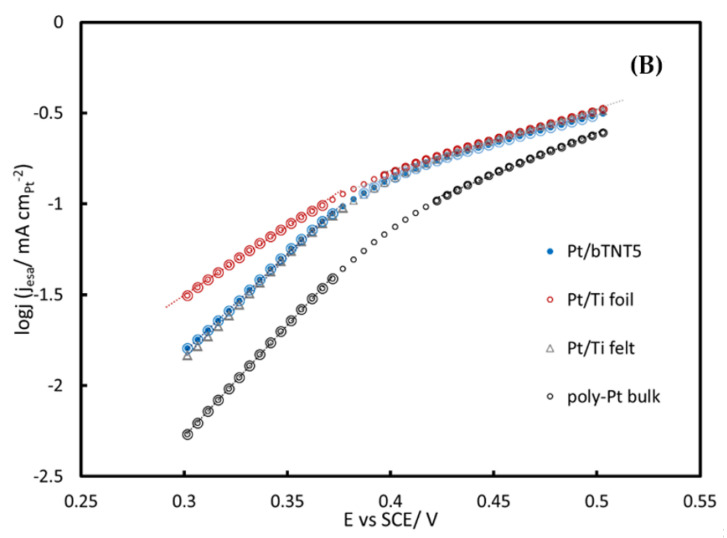
(**A**) Voltammograms of Pt/bTNT5, Pt/Ti foil, Pt/Ti felt and polycrystalline bulk Pt in a N_2_-deaerated solution of 0.1 M HClO_4_ + 0.5 M MeOH, at a low scan rate of 5 mV s^−1^, scanned between +0.1–+0.8 V_SCE_ (forward scans) and (**B**)**.** Tafel slopes of logj vs. E. The current is normalized per Pt electroactive area in cm^2^.

**Figure 7:**
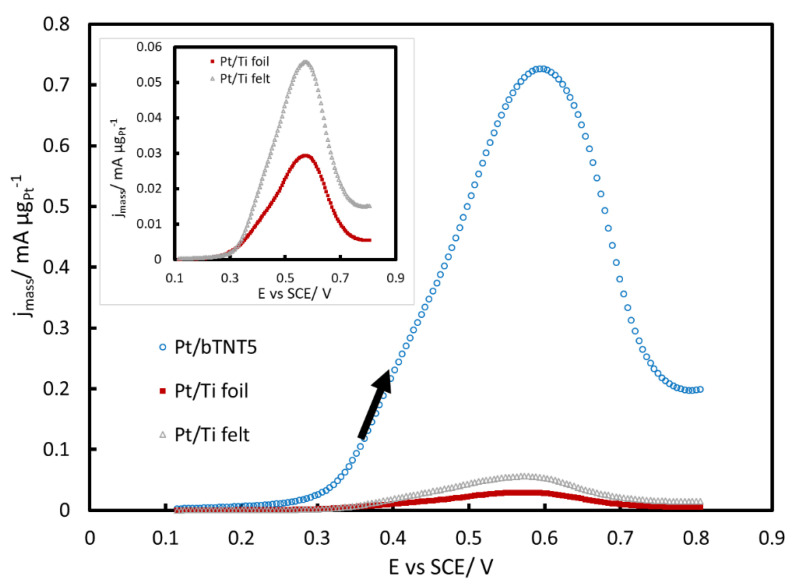
Voltammograms of Pt/bTNT5, Pt/Ti foil and Pt/Ti felt in a N_2_-deaerated solution of 0.1 M HClO_4_ + 0.5 M MeOH, at a low scan rate of 5 mV s^−1^, scanned between +0.1–+0.8 V_SCE_ (forward scans). The current is normalized per Pt mass in μg. Inset; zoom at platinized Ti foil and felt.

Figure 5, Figure 6 and Figure 7 show the near-steady state voltammograms (during both anodic and cathodic scans, as indicated by the arrows in the case of Figure 5) corresponding to methanol oxidation on platinized/platinum electrodes.

It follows from Figure 5 (presenting currents per nominal electrode substrate geometric area) that both the shape and the position of the methanol oxidation peaks is similar for all electrodes irrespective of the substrate and in line with the well established picture of methanol oxidation at Pt electrodes (see for example [56,57] and the Discussion section below), indicating that the latter does not affect the overall mechanism and that subtle oxidation rate changes should be traced at lower than the peak potentials (which is also the potential range relevant to the use of methanol in fuel cells).

Figure 6A presents results from the same near-steady-state voltammetric experiments (anodic scan) with currents normalized per Pt electroactive area (as estimated by H UPD desorption), and hence are indicative of electrocatalyst intrinsic activity (i.e., correcting for catalyst mass and surface area differences). All platinized Ti-supported electrocatalysts almost coincide and show superiority over plain Pt methanol oxidation activity, indicating a favorable Pt–Ti interaction. It is interesting to note that reduced/black TiO_2_ nanotubes show similar behavior to metallic Ti, pointing to the effective reduction of the TiO_2_ precursor material.

Figure 6B presents the Tafel slopes of all platinized Ti-supported electrodes of this work. Two regions are observed in the logj-E plot: at low overpotential values Tafel slopes are estimated at 92, 132, 90 and 82 mV dec^−1^ for Pt/bTNT5, Pt/Ti foil, Pt/Ti felt and bulk Pt respectively, while at higher overpotentials Tafel slopes tend to be 200–300 mV dec^−1^. These values are in good agreement with the ones reported in the literature in acidic media. At low overpotentials, Hou et al. [58] found that smooth bulk poly-Pt shows 125 mV dec^−1^, while Gojković et al. [59] and Christensen et al. [60] found close to 90 mV dec^−1^ (93 and 95 mV dec^−1^, respectively) and Bagotzky et al. [61] found 55–80 mV dec^−1^. Finally, Gojković et al. [59] observed 136 mV dec^−1^ at low overpotentials for Pt/C electrodes.

Figure 7 presents the results obtained at the platinized electrodes normalized per Pt mass i.e., providing the mass-specific currents/activities. It can be seen that the three-dimensional support of the Pt/bTNT electrode provides a very efficient substrate for Pt dispersion and hence very high Pt mass-specific activity and utilization. For the same reason the Ti felt-supported electrode is superior to the Ti foil one.

**Figure 8:**
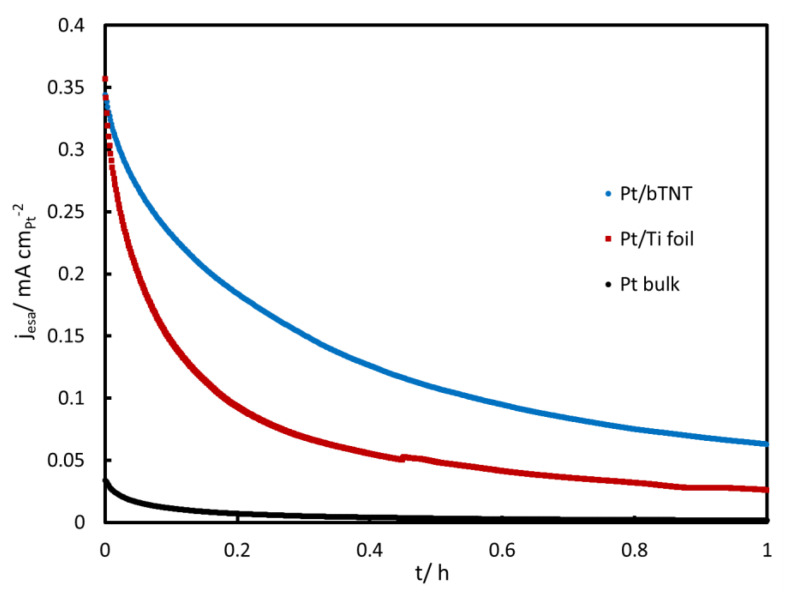
Chronoamperometric curves recorded at +0.4 V_SCE_ during 1 h. The current is normalized per Pt electroactive area in cm^2^.

Finally, Figure 8 shows a medium-term stability test at +0.4 V_SCE_ confirming that bulk Pt is contaminated very quickly, while the Pt/bTNT and Pt/Ti foil electrodes of a higher area and activity are contaminated more slowly than the bulk Pt electrode.

## 3. Discussion

### 3.1. Deposit Formation Mechanism and Morphology

Based on other similar studies [54], the mechanism of galvanic deposition of Pt on freshly etched metallic Ti substrates may occur according to the following coupled reactions:

PtCl_6_^2−^ + 4e^−^ ⇌ Pt + 6Cl^−^ +0.744 V vs. SHE (1)

TiO_2_ + 4e^−^ + 4H^+^ ⇌ Ti + 2H_2_O −1.095 V vs. SHE (2)

with the first one occurring in the forward direction (Pt reduction/deposition) and the second one in reverse (Ti oxidation/oxide formation), due to the large difference in their standard reduction potentials. In another similar study [50], it has been argued that, following TNTs treatment by CaH_2_ and its transformation to its titania black form (bTNTs), the reduced surface species on TiO_2_ are neutral oxygen vacancies ($v_{O}^{\times }$), that exist as defect complexes composed of the effective positively charged defect ($v_{O}^{••}$) and two electrons at adjacent Ti atoms. Hence, Pt galvanic deposition on bTNTs occurs via the following overall reaction:

(3) $${{{\mathrm{PtCl}}_{6}}^{2}}^{-}+2\;(2e^{-}+v_{O}^{••}{)}_{\mathrm{bTNT}}\;\to \;\mathrm{Pt}+2{\left(v_{O}^{••}\right)}_{\mathrm{bTNT}}+6\;{\mathrm{Cl}}^{-}$$

In the case of mild conditions of oxide reduction/removal (hydrogenation of TNTs/mechanical grinding of Ti foil), almost complete coverage of the outer surface/surface of the substrate by Pt is achieved by galvanic deposition (Figure 1 and Figure 2). On the contrary, upon aggressive chemical etching (HCl + NaF) of the Ti felt, a particulate, dispersed and uneven deposit was obtained (Figure 3), in accordance to that obtained for similarly prepared Ir/Ti samples [54]. This is most likely due to the higher surface area available at severely etched samples as well as the Ti surface being more reactive in the latter case so as to rapidly re-passivate/form surface oxides upon exposure to air before undergoing galvanic deposition.

### 3.2. Pt loading and Electroactive Surface Area

Based on the Pt loading measured by ICP-MS for bTNTs, Ti felt and Ti foil substrates (19, 65 and 214 μg cm^−2^, respectively) and Pt electroactive surface area as estimated by the H UPD desorption charge (30.51, 8.95 and 14.88 cm_Pt_^2^ cm_geom_^−2^, respectively), the mass specific electroactive surface area of the deposits can be estimated as 164 m^2^g^−1^ for Pt/bTNT, 14 m^2^g^−1^ for Pt/Ti felt and 7 m^2^g^−1^ for the Pt/Ti foil electrode. The former two electrodes (especially the first one) show Pt mass specific area values characteristic of fuel cell/electrolyzer catalysts and could therefore be used as reactive GDL electrodes. The latter could also be envisaged as useful Pt DSA electrodes.

### 3.3. MOR in Acid

All voltammetric curves shown in Figure 5, Figure 6 and Figure 7 show a peak during the forward-anodic scan (at ca +0.55–+0.60 V vs. SCE) that, despite having been reported to vary linearly with the square root of potential scan rate (see for example [41]), does not correspond to full mass transport control since it reflects also the change in Pt surface state from oxide-free/partially covered to oxide-covered/fully covered (see surface electrochemistry of Figure 4) and the corresponding change in MOR activity [56,57,61]. In more detail, methanol oxidation starts to occur when PtO_x_ surface species start to form (oxidizing methanol oxidation intermediates/poisonous species that are chemisorbed at nearby free Pt sites) and reaches a maximum at an intermediate surface coverage; beyond that state/potential, the rate of MOR starts to decrease as the surface is mainly covered by PtO_x_ and less reactive Pt sites are available. The hysteresis between the forward (towards higher potentials) and reverse (towards lower potentials) scan direction reflects the hysteresis between PtO_x_ anodic formation and cathodic stripping (Figure 4) as well as the higher MOR activity of freshly produced Pt free sites following the stripping of a protective PtO_x_ layer that inhibits the accumulation of poisonous carbonaceous species.

From Figure 6, it follows that the presence of Ti or/and bTNT promotes methanol oxidation; for example, at the low overpotential value corresponding to an applied potential of +0.40 V vs. SCE (which should be relevant to the operation of direct methanol fuel cells) a nearly three-fold increase of the oxidation current is observed. This can be interpreted via a change in the electron density on Pt and a weakening of poisonous Pt-CO bonding [31,32,33,34,35,36,37,38,39,40,41,42,43,44,45,46,47,48] or the bifunctional mechanism known to operate when an oxophilic second metal (e.g., Ru, Sn, Ti, etc.) is present [27,45,49].

The origin of the current peaks, their hysteresis and the effect of Ti on MOR can all be explained taking into account the mechanism of the reaction [49]:

*Oxidative chemisorption/dehydrogenation*
  Pt + CH_3_OH → Pt-CH_3_O_ads_ + H^+^ + e^−^ (4)
  Pt-CH_3_O_ads_ → Pt-CHO_ads_ + 2H^+^ + 2e^−^ (5)
*Poison formation*
  Pt-CHO_ads_ → Pt-CO_ads_ + H^+^ + e^−^ (6)
*Reactive path (where M denotes Pt or second metal or reduced metal oxide)*
  M + H_2_O → M-OH_ads_ + H^+^ + e^−^ (7)
  Pt-CHO_ads_ + M-OH_ads_ → Pt-COOH_ads_ + M + H^+^ + e^−^ (8)
  Pt-COOH_ads_ → Pt + CO_2_ + H^+^ + e^−^ (9)
*Poison removal*
  M + H_2_O → M-OH_ads_ + H^+^ + e^−^ (10)
  Pt-CO_ads_ + M-OH_ads_ → Pt + M + CO_2_ + H^+^ + e^−^ (11)

In the case of this study, M (apart from Pt) can either be Ti or defective native Ti surface oxides or reduced bTNTs sites, whereby the following reactions can be written, respectively:

Ti + H_2_O → Ti−OH_ads_ + H^+^ + e^−^ (12)

TiO_2−x_ + H_2_O → TiO_2-x_−OH_ads_ + H^+^ + e^−^ (13)

(14) $$(2e^{-}+v_{O}^{••}{)}_{\mathrm{bTNT}}+2H_{2}O\;\to \;(2e^{-}+v_{O}^{••}{)}_{\mathrm{bTNT}}-(2{\mathrm{OH}}_{\mathrm{ads}})+2H^{+}+2e^{-}$$

Table 1 below presents a summary of MOR peak current densities recorded during voltammetric experiments at various Pt-based and Pt/Ti-based electrodes in the literature, after correcting for differences in methanol concentration and scan rate (both have been reported to be square root dependencies [41,61]).

The j_esa_ values point to the platinized Ti electrodes of this work having comparable or better intrinsic MOR catalytic activity to other Pt/C, Pt/TiO_2_ and Pt/Ti materials reported in the literature, indicating strong Pt–Ti interactions as well as extensive inter-dispersion. Both effects originate from the galvanic deposition method i.e., the formation of thin deposits (as the reaction is self-terminated) and of adjacent Pt and TiO_2_ sites (since Pt deposition/reduction of Pt(IV) is coupled locally to Ti oxidation).

The j_m_ values indicate that the Pt/bTNT electrodes show very high mass specific activity towards MOR, even higher than that of commercial Pt/C powder catalysts, whereas that of Pt/Ti felt electrodes are comparable or better than that of most Ti-supported electrodes tabulated.

## 4. Materials and Methods

### 4.1. Electrodes Preparation

The galvanic deposition method was used to deposit Pt on different Ti-based electrode substrates. Three different types of titanium material were used: conductive black titania nanotubes (bTNTs), titanium foil (Ti foil) and titanium felt (Ti felt). bTNTs and plasma-etched Ti felt were synthesized as described in the work of Liu et al. [68] and Tsampas et al. [69], respectively. (Note that bTNT5 denotes Ti anodization time of 5 min, corresponding to 2 μm long TiO_2_ nanotubes). These materials underwent different pre-treatment before Pt deposition: (A) TNTs were thermally treated at 500 ^o^ C in an ampule containing CaH_2_ to maintain a reducing atmosphere [68] and remained in the ampule until the galvanic deposition of Pt; (B) Ti foil (0.2 mm thickness, 99.5%, Alfa Aesar) was intensely grinded with a dry emery paper (80-grit) to remove surface-native oxides, increase surface roughness and enhance Pt adhesion on Ti; and, lastly, (C) the plasma-etched Ti felt was chemically etched in a boiling concentrated HCl solution with 0.14 M NaF (HCl; ChemLab 37% for laboratory use, NaF; Merck 99% for analysis) for 40 s (this was used as an alternative to foil pretreatment, as the felt could not be grinded). The fully dry and reduced bTNTs, freshly grinded Ti foil and freshly etched Ti felt were immediately immersed in a N_2_-deaerated solution of 0.5 mM K_2_PtCl_6_ + 0.1 M HClO_4_ at 65 °C for 15 min and the galvanic deposition of Pt took place spontaneously at open circuit potential. In order to prevent the competing O_2_ reduction (instead of Pt(IV)), a N_2_-blanket gas passed above the cell.

### 4.2. Microscopy and Spectroscopy

Scanning Electron Microscopy/Energy Dispersive Spectroscopy (SEM/EDS) analysis was carried out to study the morphology and the atomic composition of the Pt-based Ti-type electrodes. A JEOL 6300 SEM microscope equipped with an Oxford ISIS 2000 X-ray EDS (EDAX) system, a JEOL JSM-7610F Plus supported by an Oxford AZTEC ENERGY ADVANCED X-act energy dispersive X-ray spectroscopy system and a Hitachi SU8230 with an acceleration voltage of 3 kV equipped with EDS were used. Inductively Coupled Plasma-Mass Spectroscopy (ICP-MS) analysis was carried out to estimate Pt quantity/mass that has been spontaneously deposited on the Ti supports. Pt-based electrodes were dissolved in boiling aqua regia (37% HCl by ChemLab, 65% HNO_3_ by ChemLab) for 30 min and the leachates were diluted in 2% HNO_3_ to be analyzed at a Thermo Scientific iCAP Q ICP-MS controlled via Q Tegra software.

### 4.3. Electrochemical Setup

Autolab PGSTAT302N (Eco Chemie, Utrecht, The Netherlands) workstation controlled via NOVA 1.11.2 software was used to perform all electrochemical measurements. The working electrode was placed in the center of a three-compartment glass cell and its potential was controlled by a KCl-Saturated Calomel Electrode (SCE), which were in close distance via a Luggin capillary. A Pt foil was used as counter electrode. All potential values are referred vs. SCE (−0.303 V_SCE_ in 0.1 M HClO_4_ corresponds to 0 V_RHE_).

Surface electrochemistry of the Pt/bTNT5, Pt/Ti foil and Pt/Ti felt electrodes was studied by the cyclic voltammetry technique performed at a scan rate of 50 mV s^−1^ in a N_2_-deaerated 0.1 M HClO_4_ (70%, Merck, Darmstadt, Germany) solution between hydrogen and oxygen evolution reactions (namely −0.30–+1.20 V_SCE_). In addition, a bulk Pt electrode was scanned at 500 mV s^−1^ in the same conditions.

The methanol oxidation reaction (MOR) was studied also by the cyclic voltammetry technique at low scan rate of 5 mV s^−1^ (near-steady-state) at a potential window of +0.1–+0.8 V_SCE_ in a N_2_-deaerated solution of 0.1 M HClO_4_ + 0.5 M MeOH.

## 5. Conclusions

Galvanic deposition of Pt from its chloro-complex Pt(IV) solution occurs spontaneously on freshly reduced/etched Ti substrates irrespective of the form (TNTs, felts, foils) and pretreatment (chemical reduction, chemical etching, grinding).

The presence of a Ti or bTNT substrate increased the intrinsic MOR activity of the Pt catalyst in acid.

Pt/bTNT/Ti electrodes exhibited a very high mass specific activity towards MOR (ca 700 mA mg_Pt_^−1^ at the voltammetric peak of 5 mVs^−1^ in 0.5 M MeOH) making the material ideal as a MOR catalyst in DMFCs and electrolyzers (if the Pt/bTNTs are removed from the Ti support, e.g., by ultra-sonication, and applied on the MEA of a PEM fuel cell or electrolyzer).

Pt/Ti felt electrodes exhibited a good MOR mass specific activity (ca 60 mA mg_Pt_^−1^ at the voltammetric peak of 5 mVs^−1^ in 0.5 M MeOH), making them good candidates for reactive GDLEs in gas feed DMFCs and electrolyzers.

## Figures and Tables

**Table 1:** MOR voltammetric peak current densities referred to methanol concentration of 0.5 M and potential scan rate of 5 mV s^−1^.

Reference	Electrode	j_m_/mAmg^−1^	j_esa_/mAcm^−2^_Pt_
Hayden 2001 [62]	Pt/TiO_2_	1.41	0.14
Momeni 2015 [63]	(Pt + MWCNT)/Ti	34.23	
Fan 2013 [64]	Pt/TiO_2_ + C	31.62	0.35
Song 2011 [65]	Pt/TNT	3.49	
Yang 2009 [37]	Pt/TNT + C	13.12	
Wang 2011 [39]	Pt/TNT/Ti	8.62	
Sui 2014 [40]	Pt/TNT + C	117.00	
Han 2018 [43]	PtRu/graphene-TiO_2_	67.08	
Hassan 2009 [46]	Pt/Ti	6.96	0.12
Abraham 2021 [48]	Pt/Ti	18.92	
Chen 2008 [66]	Pt/C (E-Tek)	7.50	
Sui 2014 [40]	Pt/C (Vulcan XC-72)	117.00	
Wang 2021 [67]	Pt/C (JM)	395.28	0.29
this work	Pt/bTNT	726.00	0.44
this work	Pt/Ti felt	57.00	0.41
this work	Pt/Ti	29.00	0.42

## Data Availability

Not applicable.

## Supplementary Materials

The following are available online at https://www.mdpi.com/article/10.3390/molecules27196382/s1, Figure S1: X-ray diffractogram of Pt/bTNT that includes peaks from TiO_2_ in anatase structure, hexagonal Ti and cubic Pt according to the corresonding ICDD-JCPD files. As it can be observed, the Ti and Pt characteristic peaks are present in the spectrum of Pt/bTNT.

## Sample Availability

Samples of plasma-etched Ti felt are available from the authors.
