# Supplementary material for: Methanol Oxidation at Platinum Coated Black Titania Nanotubes and Titanium Felt Electrodes
Source: Molecules. 2022 Sep 27;27(19):6382. doi: 10.3390/molecules27196382 (PMC9571902; doi:10.3390/molecules27196382)
Supplement: Supplementary file 1 [file molecules-27-06382-s001.zip › molecules-1825930-supplementary.pdf]

SUPPORTING INFORMATION

Methanol oxidation at platinum coated black titania nanotubes and titanium felt electrodes

Aikaterini Touni [1], Xin Liu [2], Xiaolan Kang [2], Chrysanthi Papoulia [3], Eleni Pavlidou [3], Dimitra Lambropoulou [1], Mihalis N. Tsampas [4], Athanasios Chatzitakis [2] and Sotiris Sotiropoulos [1,*]

1 Department of Chemistry, Aristotle University of Thessaloniki, 54124, Greece
2 Centre for Materials Science and Nanotechnology, Department of Chemistry, University of Oslo, Gaustadalléen 21, NO-0349 Oslo, Norway
3 Department of Physics, Aristotle University of Thessaloniki, 54124, Greece
4 Dutch Institute for Fundamental Energy Research (DIFFER), 5612 AJ Eindhoven, the Netherlands
* Corresponding author: Sotirios Sotiropoulos, eczss@chem.auth.gr

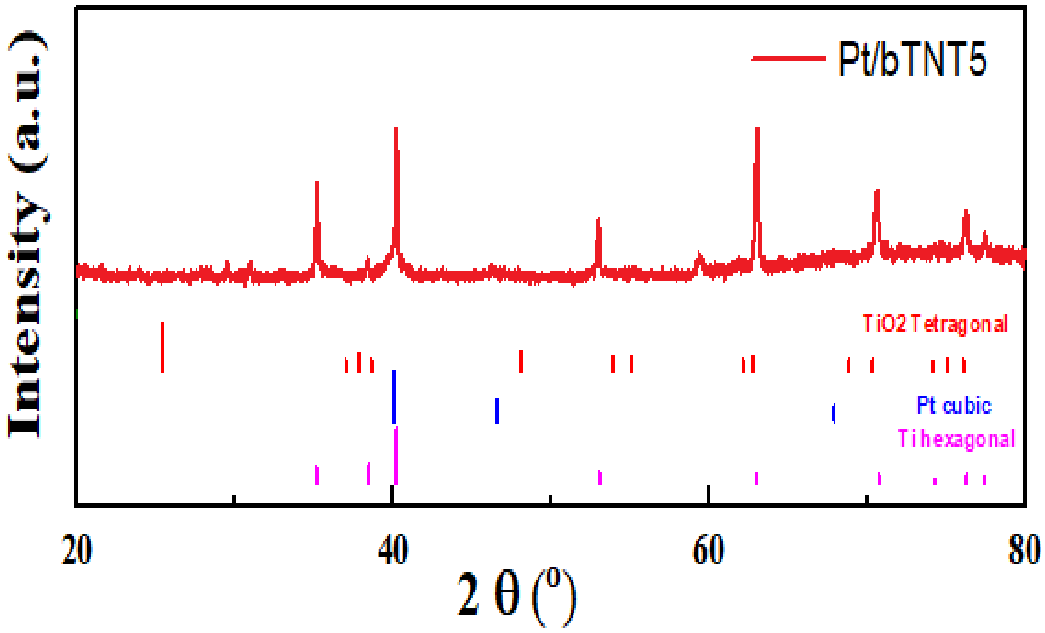

Figure SI.1 X-ray diffractogram of Pt/bTNT that includes peaks from TiO_2 in anatase structure, hexagonal Ti and cubic Pt according to the corresonding ICDD-JCPD files. As it can be observed, the Ti and Pt characteristic peaks are present in the spectrum of Pt/bTNT.
